# Aberrant CD40-Induced NF-κB Activation in Human Lupus B Lymphocytes

**DOI:** 10.1371/journal.pone.0041644

**Published:** 2012-08-27

**Authors:** Wen Zhang, Qun Shi, Xiaotian Xu, Hua Chen, Wei Lin, Fengchun Zhang, Xiaofeng Zeng, Xuan Zhang, Denian Ba, Wei He

**Affiliations:** 1 Department of Rheumatology, Peking Union Medical College Hospital, Peking Union Medical College and Chinese Academy of Medical Science, Beijing, China; 2 Department of Immunology, School of Basic Medicine, Peking Union Medical College and Institute of Basic Medical Sciences, Chinese Academy of Medical Sciences, No. 5, Dong Dan San Tiao, Beijing, China; University of Ottawa, Canada

## Abstract

Auto-reactive B lymphocytes and its abnormal CD40 signaling play important roles in the pathogenesis of systemic lupus erythematosus (SLE). In this study, we analyzed CD40 expression and CD40/CD154 induced activation of NF-κB signaling pathway in B cells from SLE patients. B cells from healthy volunteers and tonsilar B cells from chronic tonsillitis were used as negative and positive controls. Results showed CD40-induced NF-κB signaling was constitutively activated in B cells from active lupus patients, including decreased CD40 in raft portion, increased phosphorylation and degradation of IκBα, phosphorylation of P65, as well as increased nuclear translocation of P65, P50, c-Rel, which could be blocked by anti-CD154. CD154 stimulation could induce further phosphorylation and degradation of IκBα, as well as phosphorylation of P65 and nuclear translocation of P65. In addition, CD40-induced kinase activities in B cells from lupus patients mimicked that of tonsil B cells, in that IKKα/β were more activated compared to normal B cells. CD40-induced NF-κB activity was blocked by both IκB phosphorylation and proteosome degradation inhibitors in both lupus and normal B cells. All together, our findings revealed that canonical NF-κB signaling is constitutively activated in active lupus and is mediated by CD154/CD40. CD40 induced NF-κB activation is different in human lupus B lymphocytes compared with normal B cells.

## Introduction

Systemic lupus erythematosus (SLE) is a complex autoimmune disease which is characterized by abnormal B cell activation and differentiation into short and long lived plasma cells that produce pathogenic autoantibodies [1]. Although the pathogenesis of SLE is not yet fully understood, considerable evidence supports that B-lymphocytic loss of tolerance to autoantigen leads to subsequent disease progression [2].

CD40 is a TNF superfamily transmembrane glycoprotein expressed mainly on B cells, and it plays a pivotal role in B cell differentiation and activation [3]. When activated, CD40 can recruit scaffolding proteins such as TRAFs (TNF receptor-associated factor) to bind with its intracellular domains and activate downstream NF-κB pathways [4]–[6]. Interaction between CD40 and its ligand, CD154, provides a costimulatory signal that induces T cell-dependent B cell proliferation and differentiation with subsequent antibody production, which plays an important role in the pathogenesis of SLE [7]–[9]. Early in 1996, Koshy M et al found increased expression of CD154 on SLE lymphocytes [10]. In 1999, Vakkalanka RK et al demonstrated high level of serum CD154 in most lupus patients, and CD154 had the capacity to mediate B cell apoptosis by inducing CD95 expression [11]. In addition, serum CD154 level correlated with the titer of anti-double-stranded DNA antibody and with disease activity [12]. Meanwhile, CD40 expression was found up-regulated in kidney of lupus nephritis [13], as well as in the skin lesion of patients with subacute cutaneous lupus erythematosus [14], whereas, there was no significant difference between SLE and normal controls in CD40 expression on peripheral blood B cells [15]. Moreover, recombinant CD154-leucine zipper fusion protein could significantly increase the production of total IgG and autoantibodies by SLE B cells [15]. When antagonistic anti-CD154 was added in vitro, it could reduce abnormal proliferation as well as IgM and IgG secretion by peripheral B cells from lupus patients [16]. Furthermore, Studies also suggested monoclonal antibody against CD154 was a potential candidate treatment for SLE [17], [18], when SLE patients were treated with humanized anti-CD154mAb, their serum anti-dsDNA level, total amount of proteinuria and SLE disease activity were reduced [19].

However, the details of how CD40/CD154 activates NF-κB signaling pathway in SLE is unclear, and whether it is different from normal B cells remains unknown. In this study, we investigated the role of CD40 in inducing the activation of NF-κB signaling pathway in peripheral B lymphocytes from SLE patients, and compared with B cells from normal controls and tonsils.

## Results

### Constitutive activation of canonical NF-κB signaling in peripheral B cells from active SLE patients

**Figure 1:**
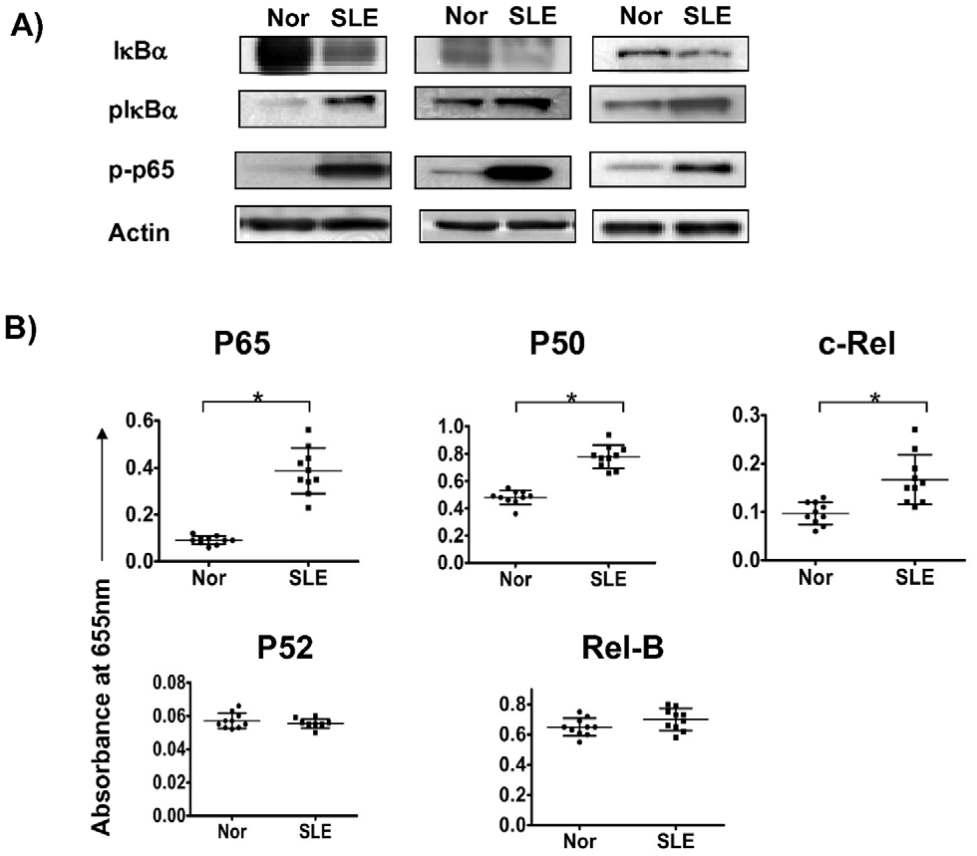
Constitutive expression of canonical NF-κB signaling in SLE patients. Isolated primary B cells were lysed and whole cell extractions were generated and then examined by western blot. Canonical NF-κB signaling was shown by phosphorylation and degradation of IκBα, phosphorylation of P65 (Fig. 1A); as well as nuclear translocation of P65, P50, c-Rel, Rel-B and P52 (Fig. 1B) (Y axis represents absorbance at 655 nm). Fig. 1A showed the results of three blots performed on three different controls and three different lupus patients. In Fig. 1B, data are depicted as the mean value± SD of ten independent experiments performed on ten different controls and ten different lupus patients. Statistical significance is determined by the one-tailed paired two-sample Student's t test and is indicated as follows: *p≤0.05).

In order to investigate whether *NF-κB* signaling is constitutively activated, the expression of endogenous NF-κB signaling subunits (IκBα, pIκBα, IκBβ, IκBδ, P65 and pP65) were measured and compared between B cells from normal controls and SLE patients. Isolated primary B cells were lysed and whole cell extractions were generated and then examined by western blot. The result revealed that canonical NF-κB signaling was spontaneously activated in peripheral B cells from active SLE patients compared with normal B cells, as shown by increased phosphorylation and degradation of IκBα, phosphorylation of P65 (Fig. 1A), but not IκBβ or IκBδ(data not shown). The fold increase of relative band densities of B cells from lupus patients to normal counterparts in pP65, pIκBα and IκBα were 5.2±1.6, 1.7±0.4, and 0.34±0.07, respectively (p<0.05). In addition, nuclear translocation and DNA binding of NF-κB subunits was tested by ELISA-based Transfactor Assay on extracted nuclear proteins from B cells, which revealed increased nuclear translocations of P65, P50 and c-Rel, but not Rel-B and P52 (Fig. 1B), the fold increase of above NF-κB subunits compared with normal controls were 4.33±1.34 (p<0.05), 1.63±0.14 (p<0.05), 1.93±1.04 (p<0.05), 1.08±0.15 (p>0.05) and 0.97±0.07 (p>0.05) respectively. There was no significant correlation between the level of nuclear P65, P50 and c-Rel and the titre of anti-dsDNA and proteinuria (p>0.05).

### CD40 expression was decreased in raft portion in peripheral B cells from SLE patients

**Figure 2:**
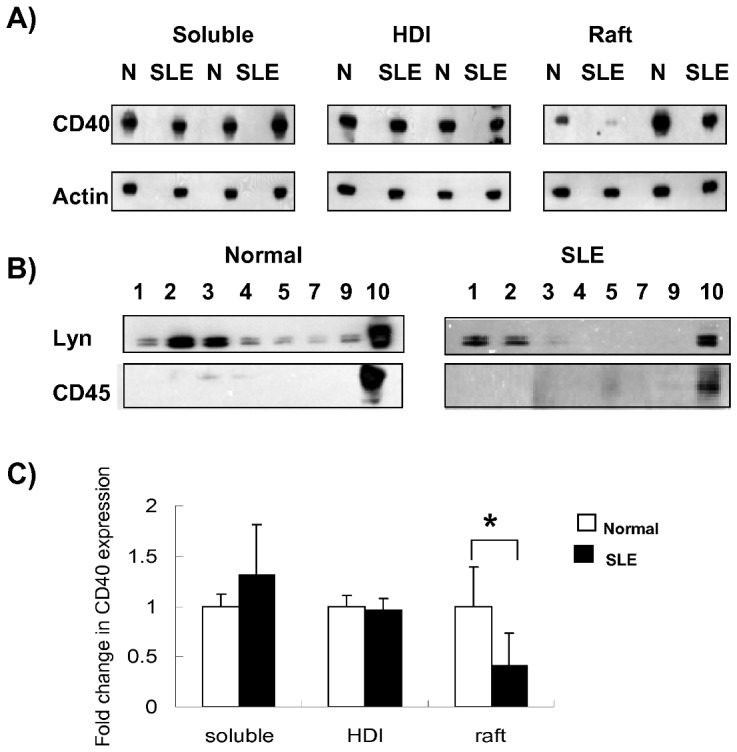
CD40 expression in raft, soluble, and high-density insoluble (HDI) portion of peripheral B cells. Peripheral CD19 +B cell were sorted. Cell lysates were then separated into raft portion, soluble portion and high-density insoluble portion (HDI). CD40 expression in each portion was examined with western blot (Fig. 2A). The purity of each portion was confirmed by western blot with anti-lyn and anti-CD45 antibodies (Fig. 2B). Statistical analysis of relative band densities was shown in Fig. 2C (the bars represents the mean value of all fractions of each portion). Significance is determined by the one-tailed paired two-sample Student's t test and is indicated as follows: *p≤0.05.

As described above, NF-κB signals were constitutively activated in primary B cells from SLE patients. According to literature, CD40 engagement contributes to activation of NF-κB signaling in B cells, it would be interesting to see whether in B cells from lupus patients, the constitutive activation of NF-κB pathway was induced in association with CD40 signaling. We next tested the expression of CD40 in cell surface as well as in soluble portion, high density insoluble portion (HDI) and raft portion in lupus and normal B cells. Total surface expression of CD40 was measured with flow-cytometry, while CD40 expression in each portion was examined with western blot after extraction of different portions from B cells. The results showed that although there was no significant difference in CD40 expression on peripheral blood B cells between SLE and normal controls (data not shown), we found that primary B cells from lupus patients had less CD40 in raft portion but not in soluble portion and HDI portion (Fig. 2A,B). The relative band density of CD40 in raft from B cells in normal controls and lupus patients were 1.0±0.39 vs. 0.41±0.28, *p*<0.05 (Fig. 2C), suggesting CD40 underwent degradation in raft portion in peripheral B cells from active SLE patients.

### CD40 induced activation of NF-κB pathway in peripheral B cells was different between normal controls and SLE patients

**Figure 3:**
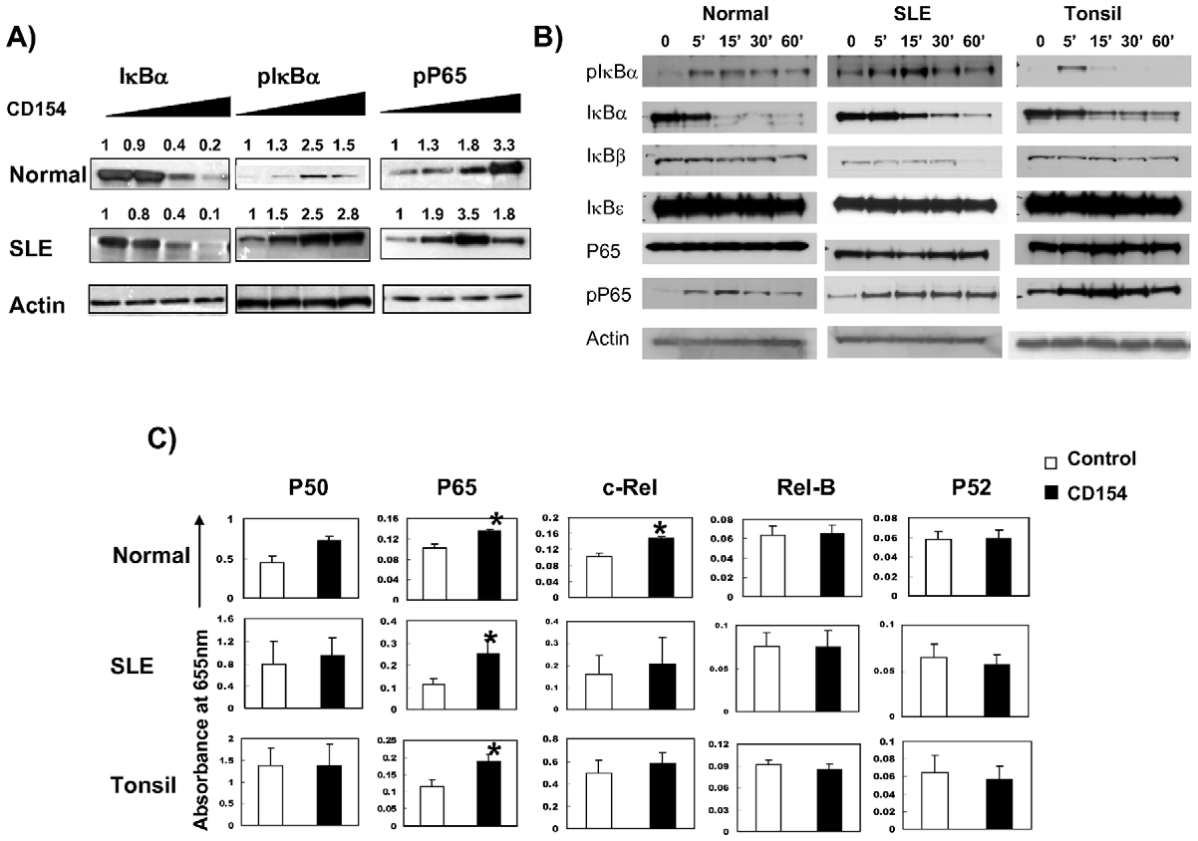
CD40 is involved in activation of NF-κB pathway in peripheral B cells from normal controls, SLE patients and tonsils. Peripheral B cells from SLE patients and normal controls were incubated with different concentrations of CD154 (0, 0.005 ug/100 K cells, 0.05 ug/100 K cells, 0.5 ug/100 K cells), or stimulated with CD154 (0.125 ug/100 K) at different time points (0, 5, 15, 30, 60 minutes). The expressions of pIκBα, IκBα, IκBβ, IκBε, P65, and pP65 from whole cell extraction were tested by western blot (Fig. 3A, B). Results from one of five independent experiments are shown; then, nuclear proteins were isolated and assessed for NF-κB binding activity with NF-κB transfactor assays specific for P65, P50, p52, c-Rel and Rel-B (Fig. 3C). Nuclear translocation data are depicted as the mean value± SD. Y axis represents absorbance at 655 nm. Statistical significance is determined by the one-tailed paired two-sample Student's t test and is indicated as follows: *p≤0.05.

Next we investigated if CD40 stimulation could induce further activation of NF-κB signaling in B cells from lupus patients. CD154-induced NF-κB signaling was measured with different concentrations of CD154 and at different time points. We found that the peak P65 phosphorylation level in B cells from lupus patients was achieved with less CD154 compared with that in normal B cells (Fig. 3A), suggesting that B cells from lupus patients are more sensitive to CD154 stimulation. Similar to that in tonsil B cells, CD154 stimulation could induce further NF-κB activation in B cells from lupus patients, including phosphorylation and degradation of IκB-α and phosphorylation of P65 (Fig. 3B), as well as nuclear translocation of P65 but not P50 or c-Rel (Fig. 3C).

### Antagonistic anti-CD154 could block baseline nuclear translocation of P65 and c-Rel, as well as phosphorylation of P65 and IκBα in active B cells from lupus patients

**Figure 4:**
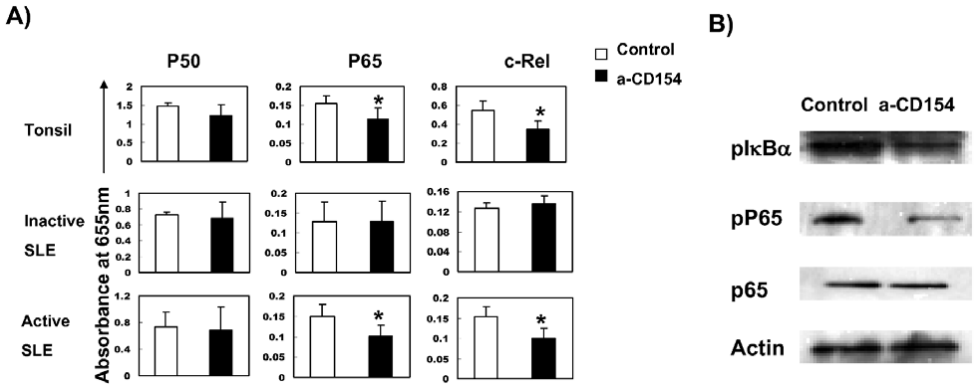
Effects of antagonistic anti-CD154 on nuclear translocation of P50, P65 and c-Rel as well as phosphorylation of P65 and IκBα in B cells from active lupus patients and tonsil B cells. B cells from active, non-active lupus patients and tonsil B cells were incubated in the presence or absence of 10 µg/ml TRAP1 (anti-CD154) for 2 hours. Nuclear proteins were isolated and assessed for NF-κB binding activity of P50, RelA/P65, and c-Rel by NF-κB transfactor assays (Fig. 4A). Y axis represents absorbance at 655 nm. Data are depicted as the mean value± SD. Statistical significance is determined by the one-tailed paired two-sample Student's t test and is indicated as follows: *p≤0.05. Whole cell lysate was tested for phosphorylation of P65 and IκBα in B cells from active SLE patients by western blot (Fig. 4B). Results from one of five independent experiments are shown.

In above experiments, we confirmed that canonical NF-κB signaling was spontaneously activated in B cells from active SLE patients. We also demonstrated that CD40 expression was decreased in raft portion and engagement of CD154 induced further activation of NF-κB. As previous studies revealed that B cells from active lupus could also express CD154 both in human and mice model [16], we set out to investigate whether CD154 expressed on B cells is involved in constitutive NF-κB activation by treating B cells from lupus patients with antagonistic anti-CD154, and we found that blocking CD154 expression on B cells from active lupus patients could antagonize constitutive activation of canonical NF-κB signaling, including nuclear translocation of P65 and c-Rel, which was similar to that observed in tonsil B cells but not in B cells from non-active SLE patients (Fig. 4A). Antagonistic anti-CD154 also inhibited the phosphorylation of IκBα and P65 (Fig. 4B).

### CD40-induced kinase activities of IKKα, β, ε and TBK1 were different in B cells from lupus patients compared to normal B cells

**Figure 5:**
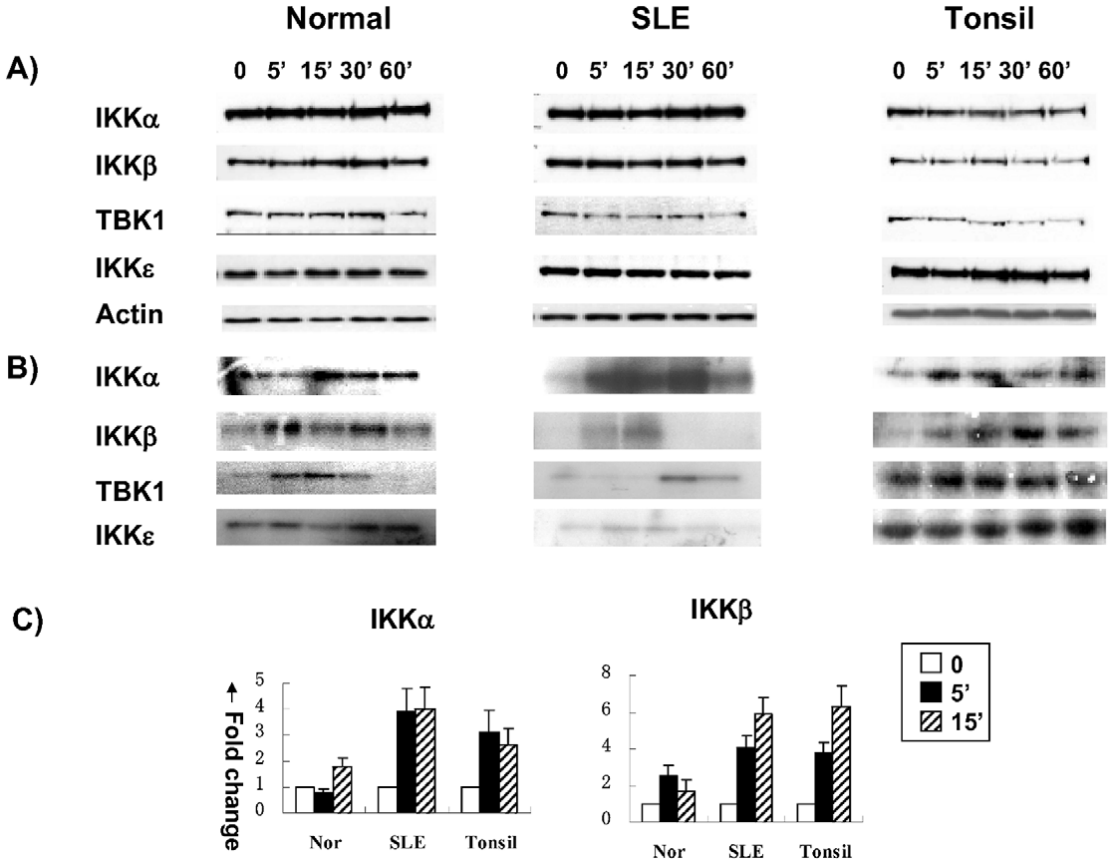
Kinase activities of IKKα, β, ε and TBK1 in normal, SLE and tonsil B cells. After stimulation with recombinant CD154 (0.125 µg/100 k cells) for 0, 5, 15, 30 or 60 minutes, kinase activities of IKKα, β, ε and TBK1 in normal, SLE and tonsil B cells were tested. 5A: Total protein levels of IKKα, β, ε &TBK1 by western blot; 5B: Kinase activities using GST-IκBα as substrate. 5C: relative kinases activity (relative band densitometry analysis) results from five independent experiments.

Next, we wanted to know which kinase upstream of IκB is involved in CD154-induced activation of NF-κB signaling. We examined the protein levels as well as upstream kinase activities of IκB including IKKα, β, ε and TBK1 in normal and B cells from lupus patients as well as tonsil B cells which were stimulated with recombinant CD154. Our results showed that the total cell protein levels of IKKα, β, ε and TBK1 were not affected by CD154 stimulation in B cells from both normal controls and lupus patients (Fig. 5A), whereas CD154-induced kinase activities were different in normal controls and lupus patients (Fig. 5B), with the later mimicked that of tonsil B cells, in that IKKα and IKKβ were more activated in SLE B cells compared to normal B cells (Fig. 5C).

### The effect of different signaling molecule inhibitors in CD40 induced NF-κB signaling in B cells from lupus patients and normal B cells

**Figure 6:**
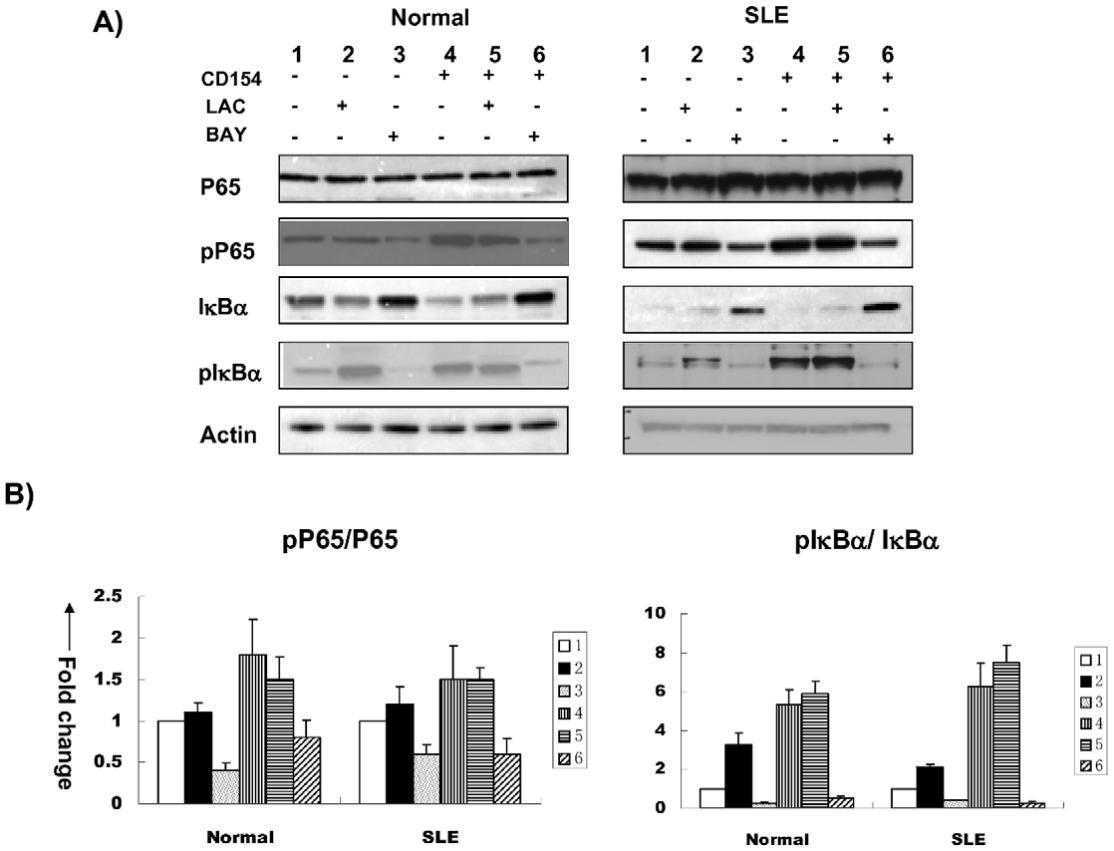
The effects of different signaling inhibitors on CD40-induced activation of NF-κB in lupus and normal B cells. IκBα, pIκBα, P65, and pP65 levels in normal and B cells from lupus patients before and after stimulation with recombinant CD154 in the presence or absence of lactacystein (LAC, 30 uM) and BAY11-7082 (BAY, 30 uM) were tested by western blot (Fig. 6). 6A: Results from one of five independent experiments. 6B: Relative band density ratio of pP65/P65 and pIκBα/IκBα in lupus and normal B cells. The relative band densities of pP65/P65 and pIκBα/IκBα with CD154 stimulation compared to no stimulation in normal controls and SLE patients were 1.8±0.43, 5.33±0.8 and 1.5±0.4, 6.25±1.23 respectively (p<0.05). For inhibition test, compared with baseline levels (no CD154, no inhibitors), the relative band densities of pP65/P65 with LAC inhibition (no CD154) and BAY(no CD154) in normal controls were 1.1±0.12 (p>0.05) and 0.4±0.09 (p<0.05), in SLE patients were 1.2±0.21 (p>0.05) and 0.6±0.11 (p<0.05); the relative band densities of pIκBα/IκBα with LAC inhibition(no CD154) and BAY (no CD154) in normal controls were 3.28±0.6 (p<0.05) and 0.27±0.06 (p<0.05), in SLE patients were 2.1±0.16 (p<0.05) and 0.39±0.04 (p<0.05). When stimulated with CD154, compared with that with CD154 stimulation but no inhibitors, the relative band densities of pP65/P65 with LAC inhibition (plus CD154) and BAY(plus CD154) in normal controls were 1.5±0.28 (p>0.05) and 0.8±0.21 (p<0.05) respectively, in SLE patients were 1.5±0.15 (p>0.05) and 0.6±0.19 (p<0.05) respectively.; the relative band densities of pIκBα/IκBα in normal controls were 5.9±0.62 (p>0.05) and 0.53±0.1 (p<0.05) respectively, in SLE patients were 7.5±0.91 (p>0.05) and 0.26±0.08 (p<0.05)respectively. There was no statistical difference between normal controls and SLE patients at each comparison (p>0.05).

Finally, we checked if other signaling molecules are involved in the cross-talking with CD40/NF-κB pathways by screening with different signaling molecule inhibitors including 30 uM LAC (proteosome degradation inhibitor), 30 uM BAY (IκB phosphorylation inhibitor). As shown in Figure 6A, inhibition of proteosome degradation with LAC and IκB phosphorylation with BAY were similarly effective in blocking CD40-induced NF-κB signaling in B cells from lupus patients and normal controls. CD40-induced degradation of IκBα was blocked by both IκB phosphorylation and proteosome degradation inhibitors, whereas CD40-induced phosphorylation of IκBα and P65 were blocked only by IκB phosphorylation inhibitor (Figure 6B).

## Discussion

In this study, we analyzed CD40 expression and CD40/CD154 induced activation of NF-κB signaling pathway in B cells from SLE patients, and we demonstrated that canonical NF-κB signaling is constitutively activated in active lupus and is mediated by CD154/CD40. CD40 induced NF-κB activation is different in human lupus B lymphocytes compared with normal B cells.

By comparison of endogenous expressions of phosphorylated IκBα, P65, degradation of IκBα and nuclear translocation of NF-κB subunits, we found that NF-κB signaling pathway was constitutively activated in peripheral B cells from active lupus patients (Fig. 1). Although the cell surface expression of CD40 in B cells from lupus patients was similar to normal controls, which was in consistent with previous study [15], our study, however, revealed that CD40 expression was reduced in raft portion in B cells from lupus patients (Fig. 2). Previous studies have demonstrated that CD40 mediates NF-κB signaling via TRAF proteins. Upon CD40 activation, TRAF2 and TRAF3 move into insoluble buoyant complexes (lipid raft) [20], [21], bind directly to CD40 cytoplasmic tail, and then are ubiquitinated and degraded by the proteosome [22], [23]. It has also been shown that TRAF2 and TRAF3 translocation into the membrane rafts is consistently induced by CD40 activation, and CD40-dependent degradation of TRAF2 is believed to account for the negative feedback on activation. Whereas, so far, there is no data on raft expression of CD40, especially in B cells from lupus patients. Our results suggested that CD40 level in raft portion was decreased in activated B cells, which was similar to TRAF2. As the raft proportion of CD40 accounts for only a small portion of total CD40, the decrease didn't make significant change of total surface expression.

Next, we explored CD154-induced NF-κB activation in B cells from lupus patients. We demonstrated that in B cells from lupus patients, CD154 stimulation induced additional activation of NF-κB signaling, including phosphorylation of P65 and IκBα, degradation of IκBα, as well as nuclear translocation of P65, but not P50 and c-Rel, and B cells from lupus patients were more sensitive to CD154 stimulation than normal B cells as the peak p65 phosphorylation was obtained with less amount of CD154 stimulation (Fig. 3).

Moreover, we found the activities of the upstream kinases in CD40/NF-κB signaling in B cells from lupus patients were also different from that in normal controls, but mimicked that of tonsil B cells. As showed in Fig. 5, CD40-induced kinase activities were different in B cells from normal controls and lupus patients, IKKα/β were more activated in SLE B cells compared to normal B cells, especially IKKα.

CD154 surface protein levels were increased in CD4 T cells from SLE patients as compared with controls, and this increase correlated with the presence of nephritis and increased CD154 transcription rates [24]. In addition, it was found that large B cells from male BXSB lupus mice model expressed functionally active CD154, and suggested that CD154 expression on B cells and increased susceptibility to CD40 signaling due to an intrinsic B cell hyperactivity which promoted autoimmune disease in BXSB lupus mice [25]. Study on human SLE also demonstrated that CD19+ peripheral B cells expressed functionally active CD154 and were downregulated after treatment with humanized anti-CD154 mAb [16]. In our study, we found that antagonistic anti-CD154 blocked baseline phosphorylation of P65 and IκBα, as well as nuclear translocation of P65 and c-Rel in B cells from active lupus patients (Fig. 4), suggesting that, in active SLE patients, B cells express functionally activated CD154 and are involved in constitutive activation of NF-κB signaling pathway. Our data (Fig. 1B, 3C & 4A) also suggest that P65 is the major NF-κB subunit regulated by CD40 signaling.

As we known, tonsil B cells from chronic tonsillitis are in activated status, and therefore were included in this study as “positive controls”. It was intriguing to find that CD154-induced additional activation of NF-κB signaling in B cells from active lupus patients was similar to tonsil B cells. In addition, the reaction of B cells from lupus patients to anti-CD154 inhibition also mimicked that of tonsil B cells, which strongly suggested that B cells from lupus patients are in activated stage and CD40 plays similar role in activating NF-κB signaling in both SLE and tonsil B cells.

Finally, by using kinase inhibitor (BAY) and proteosome degradation inhibitor (LAC), we found that in both lupus and normal B cells, CD40-induced degradation of IκBα was blocked by both IκB phosphorylation and proteosome degradation inhibitors, whereas CD40-induced phosphorylation of IκBα and P65 were blocked only by IκB phosphorylation inhibitor (Fig. 6), suggesting CD40-induced NF-κB activation depends on both IκB phosphorylation and proteosome degradation.

In summary, we demonstrated that CD40-induced NF-κB signaling was constitutively activated in B cells from active lupus patients, including increased phosphorylation and degradation of IκBα, phosphorylation of P65, as well as increased nuclear translocation of P65, P50, c-Rel, which could be blocked by antagonistic anti-CD154. CD154 stimulation induced further phosphorylation, degradation of IκBα, phosphorylation of P65, and nuclear translocation of P65. In addition, CD40-induced kinase activities in B cells from lupus patients mimicked that of tonsil B cells. In B cells from lupus patients, NF-κB activation was mainly via CD40, and it depended both on IκB phosphorylation and proteosome degradation. Taken together, our study demonstrated NF-κB signaling is constitutively activated in active lupus and is mediated by CD154/CD40. CD40 induced NF-κB activation is different in lupus B lymphocytes compared with normal B cells.

## Materials and Methods

### Patients and controls

All patients (n = 25) involved in this study fulfilled the 1997 American College of Rheumatology classification criteria for SLE. All were female, age ranged from 18 to 45 yrs with a mean age of 32.1±8.4 yrs (Table 1). All patients were new onset treatment-naïve patients, and seventeen of them were in active disease stage, the other eight were non-active SLE patients. SLE disease activity was determined by SLE-DAI (SLE disease activity index) of which greater than 5 points indicated active disease [26]. Twenty age- and sex- matched healthy volunteers were involved as normal controls. Tonsil samples (n = 6) were obtained from non-SLE individuals (age 6 yr-23 yr) who underwent routine tonsillectomy because of chronic tonsillitis. Tonsil B cells indicated active germinal center (GC) B cells and were included as active control [27], [28]. All the patients and normal controls were of Chinese Han ethnicity. The study was approved by the ethics committee of Peking Union Medical College Hospital. Written informed consents were obtained from all patients or their relatives. Each experiment was repeated at least in five different patients.

**Table 1:** Demographic and clinical characteristics of the SLE patients (n = 25).

Female/male(n)	25/0
Age, mean ± SD (years)	32.1±8.4(18–45)
Disease duration, mean ± SD (months)	2.1±2.5(1–4)
SLEDAI, mean ± SD	10.2±5.3(2–19)
Lupus nephritis(proteinuria≥0.5 g/24 h)(n,%)	6, 24%
Neuropsychiatric manifestations (n,%)	2, 8%

### Preparation of B cells from tonsil tissue and peripheral blood

Peripheral blood mononuclear cells (PBMC) were separated by Ficoll-Hypaque density gradient centrifugation. B cells were negatively selected after staining with a mixture of dextran cross-linked mAbs specific for glycophorin A, CD2, CD3, CD14, CD16, CD33, and CD56, followed by exposure to a magnetic colloid covalently linked to anti-dextran mAb(StemCell Technologies, Vancouver, British Columbia, Canada). Tonsilar mononuclear cells were obtained as described previously [29]. Briefly, tonsils were minced and digested in RPMI containing 210 U/ml collagenase type I (Worthington Biochemical Corp., Lakewood, New Jersey, USA) and 90 U/ml DNase (Sigma-Aldrich) for 30 minutes at 37°C. Following filtration through a wire mesh, the cells were washed twice in 20% Fetal Bovine Serum (FBS)/RPMI and once in 10% FBS/RPMI before centrifugation over diatrizoate/Ficoll gradients. Tonsil B cells were negatively selected using the same technique used to isolate peripheral B cells. The purity of isolated B cells (CD19+ by flow cytometry) was>95%. Total amount of purified B cells for each experiment were 10^6^.

### Cell culture, CD154 stimulation, and CD154 antagonist inhibition

Negatively selected peripheral B cells were cultured in RPMI with 10%FBS and antibiotics (100 u/ml penicillin and 100 u/ml streptomycin). Cells were stimulated with different concentration of CD154 (0, 0.005 ug/100 K cells, 0.05 ug/100 K cells, 0.5 ug/100 K cells, eBioscience). Based on our time-point experiments, to achieved the optimal effect, for Western Blot of IκB, pIκBα and pP65(phosphorylation of IκBα and P65, degradation of IκBα), the cells were stimulated for 15 minutes; for NF-κB subunit nuclear translocation test(nuclear TransFactor assay of P65, P50 and c-Rel), the cells were stimulated for 30 minutes. In some experiments, B cells were incubated in the presence or absence of TRAP1 (anti-CD154) at concentration of 10 µg/ml for 2 hours. For inhibition experiments, B cells from normal controls and lupus patients were pre-incubated with cyclohexmide (CHX) at a concentration of 10 ug/ml with or without 30 uM Lactacystin (LAC, inhibitor of proteosome degradation) (Calbiochem) and 5 uM BAY11-7082 (BAY, IκB phosphorylation inhibitor)(Calbiochem) for 30 minutes before incubation in the presence or absence of recombinant CD154 (0.125 µg/100 k cells). Cells were collected, washed three times with Phosphate Buffered Saline (PBS). For flow cytometry experiment, the cells isolated were run as fresh samples. For western blot and nuclear transfactor experiments, after stimulation or inhibition of freshly isolated B cells, the cells were then saved in pellet and stored directly at −80°C before preparing whole cell extract or nuclear extract.

### Western Blots

Cells were harvested, washed twice in ice cold PBS, and lysed in whole cell lysates by incubation for one hour in a buffer containing 20 mM HEPES (pH 7.9), 20% Glycerol, 1% Nonidet P-40, 1 mM MgCl_2_, 0.5 mM EDTA, 0.1 mM EGTA, 1 mM DTT, 1 mM phenylmethanesulfonylfluoride(PMSF) and a proteinase inhibitor cocktail (BD Biosciences, Franklin Lakes, NJ). Lysate was kept on ice and vortexed every 10 minutes for one hour before centrifugation at 13000 rpm, 4°C. Equal amounts of protein (5 ug), as determined by Bio-Rad Protein Assay were separated by SDS-PAGE (Invitrogen) and transferred to Immobilon PVDF membranes (Millipore) and were blocked with 5% dry milk in PBS containing 0.5% Tween 20 before incubation with specific antibodies against IκB -β, -ε, (Santa Cruz Biotechnologies), IκBα, IKKα, -β, -δ and -ε (BD PharMingen), pSer^32,36^IκBα (Cell Signaling, Beverly MA), P65 (Santa Cruz Biotechnologies), pSer^536^-P65 (Cell Signaling), followed by incubation with HRP-conjugated second antibody and developed using Western Lightning Chemiluminescence Reagent Plus (PerkinElmer Life Sciences, Inc.).

### Immunoprecipitation and kinase assay

Activity of IKK was determined by IKK immunocomplex kinase assay and estimated as the ability to phosphorylate glutathione-S-transferase (GST)-IκBα. Briefly, cells were lysed in NP-40 lysis buffer (150 mM NaCl, 0.5% NP-40, 50 mM Tris, PH 8.0), add IP cocktail and PMSF for 30 minutes at 4°C. Extracts were pre-cleared with protein G-sepharose beads (Amersham Biosciences, Piscataway, NJ) for 1 h at 4°C with rotation and then incubated with anti- IKK-α, -β, -ε, and TBK1 antibodies and protein G-sepharose beads overnight at 4°C with rotation. After immunoprecipitation with specific antibody-conjugated protein-G agarose beads, lysate was washed four times in coimmunoprecipitation buffer followed by two subsequent washes in kinase buffer (25 mM Hepes, pH 7.6, 25 mM beta-glycerophosphate, 15 mM MgCl2, 1 mM DTT, 0.5 mM Na_3_VO_4_, and 0.5 mM NaF). Immunoprecipitates were then incubated for 30 minutes at 30°C with kinase buffer containing 5 µCi [γ-^32^P]-ATP and 2 µg GST-IκBα before protein fractionation by SDS-PAGE and autoradiography.

### DNA Binding of NF-κB (Transfactor Assay)

DNA binding of NF-κB (P50, P65, c-Rel and p52) components was examined in nuclear lysates by the ELISA-based TransFactor Assay (Clontech and BD Biosciences). Cells were washed twice in cold phosphate-buffered saline and nuclear extracts were prepared by using TransFactor extraction kit (BD biosciences). Briefly, cells were incubated in hypotonic pre-lysis buffer (10 mM HEPES at pH 7.9, 1.5 mM MgCl_2_, 10 mM KCl, 1 mM DTT, protease inhibitor cocktail) for 15 minutes followed by disruption with rapid strokes with a narrow-gauge needle syringe (25GA) and centrifugation at 10,000 g for 20 minutes to isolate nuclei. The pellet was resuspended in nuclear extraction buffer (20 mM HEPES at pH 7.9, 1.5 mM MgCl_2_, 0.42 M NaCl, 0.2 mM EDTA, 25% glycerol, 1 mM DTT, 1 mM PMSF and protease inhibitor cocktail) and nuclei were disrupted with rapid strokes by a narrow-gauge needle syringe (25GA). The nuclear suspension was shaken gently for 30 minutes at 4°C followed by centrifugation at 20,000 g for 10 minutes at 4°C. Equivalent amounts (20 ug) of nuclear lysates were loaded into commercially available TransFactor plates that are precoated with oligonucleotides containing consensus sequences for NF-κB components and incubated for 60 minutes at 20°C. Following the manufacturer's instructions, bound transcription factors were detected with specific primary antibodies for 60 minutes, followed by three times wash with Transfactor Blocking Buffer, then incubated with HRP-conjugated secondary antibody for 30 minutes followed by four times wash with Transfactor Buffer. Finally, bound transcription factors were detected with TMB substrate. The optical densities were measured with a microtiter ELISA plate reader using a wavelength of 655 nm. HeLa nuclear extracts with or without stimulation with TNFalpha or phorbol 12-myristate 13-acetate were used as positive controls.

### Raft extraction

The method for isolating Raft fraction was described previously [30]–[32]. Briefly, peripheral CD19 +B cell were sorted and lysed in TNEV (10 mM Tris-Hcl, PH 7.5, 150 mM NaCl, 5 mM EDTA, 0.5% Triton X-100, 1 mM Na3VO4 and protease inhibitor) for 30 min. Lysates were centrifuged 11 min at 900 g to remove nuclei and large cellular debris, then the supernatants were diluted 1∶1 with 85% wt/vol sucrose in TNE. The diluted lysate were overlayed with 35% sucrose in TNE, then slowly loaded with 1 ml 5% sucrose and ultracentrifuged at 200,000 g (34,000 RPM) for 16 hr, at 4C. After centrifuge, 10 fractions (0.5 ml each) were carefully collected from top to the bottom. Fractions 1∼4 contained Lyn and are referred to as lipid raft fractions, soluble portion was in 8∼10 fractions, the bottom pellet was high-density insoluble portion (HDI). The purity of each portion was confirmed by western blot with anti-lyn and anti-CD45 antibodies, as Lyn is mainly expressed in raft and soluble part, whereas CD45 is only expressed in soluble part.
